# Decreasing incidence of pharmacologically and non-pharmacologically treated type 2 diabetes in Norway: a nationwide study

**DOI:** 10.1007/s00125-018-4681-4

**Published:** 2018-07-11

**Authors:** Paz L. D. Ruiz, Lars C. Stene, Inger J. Bakken, Siri E. Håberg, Kåre I. Birkeland, Hanne L. Gulseth

**Affiliations:** 1 Department of Chronic Diseases and Ageing, Norwegian Institute of Public Health, Post box 4404, Nydalen, 0403 Oslo, Norway; 2 Department of Endocrinology, Morbid Obesity and Preventive Medicine, Oslo University Hospital, Oslo, Norway; 3 Institute of Clinical Medicine, University of Oslo, Oslo, Norway; 4 Centre for Fertility and Health, Norwegian Institute of Public Health, Oslo, Norway; 5 Department of Transplantation Medicine, Oslo University Hospital, Oslo, Norway

**Keywords:** Incidence, Prevalence, Register-based study, Time trends, Type 2 diabetes

## Abstract

**Aims/hypothesis:**

This study aimed to examine recent time trends in the incidence and prevalence of type 2 diabetes in Norway.

**Methods:**

In this Norwegian nationwide cohort study, we linked data from national registries with prospectively collected data on diabetes medication and diabetes diagnoses for all residents in Norway aged 30 to 89 years (>3.2 million people). We analysed trends in incidence and prevalence of type 2 diabetes from 2009 to 2014 by type of treatment, sex, age, education level and place of birth.

**Results:**

During 15,463,691 person-years of follow-up from 2009 to 2014, we identified 75,496 individuals with new-onset type 2 diabetes. Of these, 36,334 (48%) were treated with blood-glucose-lowering drugs within 6 months of diagnosis. A low education level and being born in Asia, Africa or South America were significant risk factors for incident type 2 diabetes. While the prevalence of type 2 diabetes increased from 4.9% to 6.1% during the study period, the incidence decreased significantly from 609 cases per 100,000 person-years in 2009 to 398 cases per 100,000 in 2014, an annual reduction of 10.1% (95% CI −10.5, −9.6). A declining incidence was seen for both pharmacologically and non-pharmacologically treated type 2 diabetes, and in all subgroups defined by sex, age group, education level and place of birth.

**Conclusions/interpretations:**

This nationwide study shows that, despite a decreasing incidence of type 2 diabetes in Norway, the prevalence continues to rise, probably due to diagnosis at a younger age and increased longevity.

**Electronic supplementary material:**

The online version of this article (10.1007/s00125-018-4681-4) contains peer-reviewed but unedited supplementary material, which is available to authorised users.

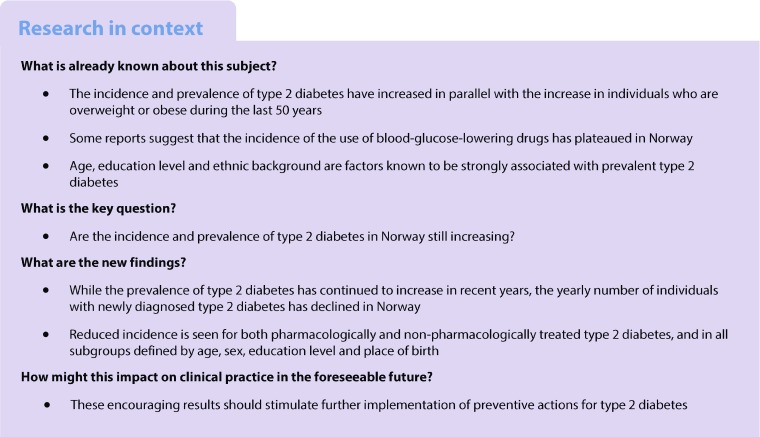



## Introduction

The prevalence of type 2 diabetes seems to be increasing in most parts of the world [1]. Prevalence of type 2 diabetes is determined by the incidence of the disease, mortality rates and other factors, such as population ageing, immigration and composition of ethnic groups, age at diagnosis, and changes in diagnostic criteria and screening activities [2]. Time trends in incidence are, therefore, potentially more informative than prevalence estimates with regards to the effects of modifiable risk factors for type 2 diabetes. Few studies report time trends in incidence of type 2 diabetes, particularly from nationally representative cohorts. Norwegian data showed no increase in incidence of non-insulin glucose lowering drug use during 2006–2011 [3]. This study, however, lacked information about the type of diabetes, non-pharmacologically treated diabetes and important demographic factors, such as immigration, ethnic background and education level.

Although the majority of people with type 2 diabetes receive pharmacological treatment [4–6], a proportion of adults with type 2 diabetes (20–40%) are omitted from analyses when only pharmacologically treated individuals are included [7–10]. Thus, differences over time in treatment patterns have not been covered by previous studies. This provides justification for the present study, which includes time trends in both pharmacologically and non-pharmacologically treated type 2 diabetes.

The complex relationship between incidence, mortality and prevalence of type 2 diabetes remains to be elucidated. In the current study, data from mandatory nationwide registries on demographic characteristics, prescription drugs, and primary and specialist healthcare visits were combined to examine recent trends in incidence and prevalence of diagnosed type 2 diabetes in Norway.

## Methods

The Regional Committee for Medical and Health Research Ethics and the Norwegian Data Protection Authority approved the study.

### Participants

In this open cohort study, we included all residents aged 30 to 89 in the period 2009 to 2014 (>3.2 million individuals). Individuals were followed from either 1 January 2009, at the age of 30 years, or 1 year after immigration to Norway, whichever occurred later. We followed individuals until diabetes diagnosis, dispense of blood-glucose-lowering medication, emigration, death, 90 years of age or the end of study period, whichever occurred first.

### Data sources

The Norwegian public health system is financed through government funding. Hospitalisations are free of charge, while there is a fee for consultations in primary care and out-of-hospital visits. We used individual-level data from three national databases (the Norwegian Prescription Database, the Norwegian Patient Registry [NPR] and the primary care database). Data were linked by means of the personal identification number unique to every Norwegian resident.

The Norwegian Prescription Database was established in 2004 and holds information on all drugs dispensed by Norwegian pharmacies. Blood-glucose-lowering drug prescriptions are classified according to the Anatomical Therapeutic Chemical (ATC) classification system in group A10 (‘Drugs used in diabetes’). In the present study, we collected information about dates and details of all glucose-lowering drugs dispensed from Norwegian pharmacies from 2004 to 2014. We included insulins and analogues classified as A10A (‘Insulins and analogues’), and non-insulin glucose-lowering medications, classified in A10B (‘Blood glucose lowering drugs, excl. insulins’).

The NPR is an administrative database covering all hospitalisations and specialist healthcare outpatient contacts in Norway, with linkable data from 2008 onwards. Diabetes diagnoses are reported according to the International Classification of Diseases, version 10 (ICD-10, www.who.int/classifications/icd/en/), in the group E10–E14 (‘Diabetes mellitus’). We collected information on date of first diagnosis and the number of times individuals were registered with these diagnoses. In the primary care database, diabetes was reported from 2006 according to The International Classification of Primary Care, Second Edition (ICPC-2; www.who.int/classifications/icd/adaptations/icpc2/en/) codes T89 (‘Diabetes insulin dependent’) and T90 (‘Diabetes non-insulin dependent’). Reporting to these health registries is compulsory and linked to the reimbursement system, with nearly complete coverage of the population (ESM Fig. 1).

## Type 2 diabetes case definition

We defined type 2 diabetes from register-based data as at least one registration of type 2 diabetes diagnosis and use of non-insulin glucose-lowering drugs, or two registrations if individuals were not treated with non-insulin glucose-lowering drugs (either E11 diagnosis code from specialist care (ICD-10) or T90 diagnosis code from the primary care database [ICPC-2]).

### Case definition of prevalent type 2 diabetes

Prevalent type 2 diabetes cases were defined as having at least one E11 or T90 diagnosis of type 2 diabetes and one or more prescriptions of non-insulin glucose-lowering drugs, or being registered with a type 2 diabetes diagnosis on at least at two different occasions. For each calendar year from 2009 to 2014, we defined the period prevalence as the number of people defined as having type 2 diabetes in that year or earlier, divided by the total number of individuals alive, aged 30 to 89 years and residing in Norway during the same calendar year (ESM Fig. 2). The end of follow-up was 31 December 2014.

### Case definition of incident type 2 diabetes

To avoid any prevalent cases of type 2 diabetes at baseline, we excluded individuals with a diagnosis of any type of diabetes or who used any blood-glucose-lowering drugs before the study start on 1 January 2009. We defined incident cases of pharmacologically treated type 2 diabetes as having the first occurrence of diabetes diagnosis in primary or specialist care and one or more prescriptions of non-insulin glucose-lowering drugs within 6 months of diagnosis. Incident cases of non-pharmacologically treated diabetes were defined as no registrations of glucose-lowering drugs and having been registered with a diagnosis of type 2 diabetes (in primary and/or specialist care) on at least two different occasions (ESM Fig. 3). The end of follow-up was 30 June 2014. Data are available until 31 December 2014, but to allow ascertainment for 6 months with or without A10 medication in the incidence analysis, we stopped follow-up time 6 months before the end of the study period. Of all incident cases, 39% were recorded in the three health registers, 36% in two registers and 26% in one register with at least two registrations of a type-2-diabetes diagnosis (ESM Fig. 4).

### Covariates

Information on sex and dates of birth, emigration and death were obtained from the National Registry [11]. Statistics Norway provided information on immigration, place of birth and education level [12]. We had information on the highest education level achieved in year 2013. Place of birth was categorised in seven broad categories: Norway, Europe except Norway, Africa, Asia, North and Central America, South America and Oceania.

### Sensitivity analysis

The number of individuals per year with possible diabetes but excluded in our algorithm (unclassified diabetes and those treated with glucose-lowering medication without a registered diagnosis of type 2 diabetes) were assessed to identify if misclassification could explain the changes in incidence trends.

### Data analysis

We estimated prevalence by counting all individuals with type 2 diabetes who were alive and aged 30–89 at some time during the given calendar year, until they emigrated or died, and dividing this number by the total number of individuals alive, aged 30 to 89 years and residing in Norway during the same calendar year (ESM Fig. 2). We estimated incidence rates with 95% CI per 100,000 person-years of follow-up, stratified by covariates such as calendar year and age group. Incidence rates were not adjusted or standardised.

Associations between risk factors and type 2 diabetes were assessed using Cox regression. We calculated annual percentage difference in incidence using Poisson regression. To assess whether the time trend in incidence changed after the date when HbA_1c_ ≥48 mmol/mol (6.5%) was recommended for diagnosing diabetes in Norway (September 2012), we used interrupted time series analysis [13]. Details of the Poisson regression models used for the latter are described in ESM Methods. Data handling and analyses were done using Stata version 15 (StataCorp, College Station, TX, USA).

## Results

### Time trends in prevalence

**Fig. 1:**
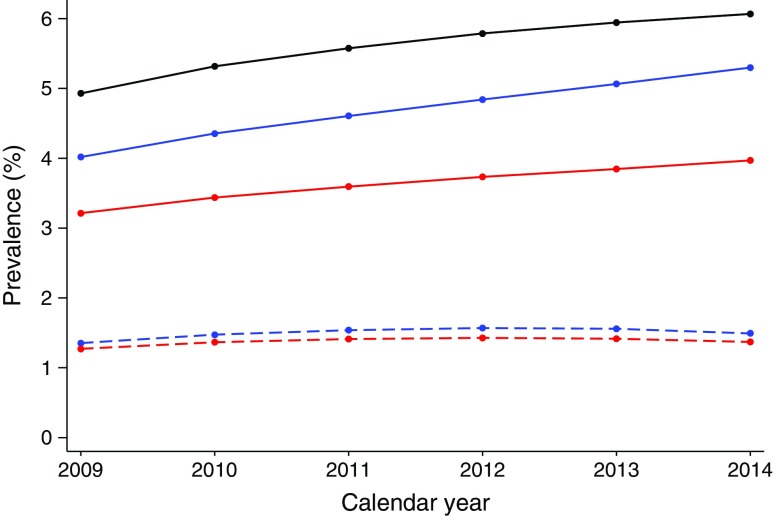
Time trends in proportion of people diagnosed with type 2 diabetes in the population aged 30–89 years in Norway from 2009 to 2014, in all participants and by treatment and sex. Black line, all participants; blue lines, men; red lines, women; solid lines, pharmacologically treated; dashed lines, non-pharmacologically treated

From 2009 to 2014, the prevalence of type 2 diabetes in Norway increased from 4.9% to 6.1% of the total population aged 30–89 (ESM Table 1). Overall, 23.6% of individuals with diabetes did not use glucose-lowering medications (Fig. 1). Type 2 diabetes was more prevalent in males than females (6.8% vs 5.3% in 2014) and the prevalence increased with age (ESM Fig. 5).

### Time trends in incidence

A total of 3,227,454 individuals aged 30–89 years were included in the analysis, with a mean follow-up of 4.8 years (range 0.002–5.5 years) (Table 1). During 15,463,691 person-years of follow-up, we identified 75,496 new cases of type 2 diabetes, corresponding to an overall incidence rate of 488.2 per 100,000 person-years (Table 2). In total, 39,162 (51.9%) of incident cases were not treated with glucose-lowering medication within 6 months of diagnosis, while 36,334 (48.1%) were treated (ESM Table 2). The mean age at diagnosis was 59.2 years for those pharmacologically treated and 62.8 years for the non-pharmacologically treated. Of the pharmacologically treated individuals, 82.6% used monotherapy metformin as their initial treatment, 4.4% used sulfonylurea monotherapy, 0.3% used other oral agents and 0.9% started treatment with insulin only (ESM Table 3). Eleven per cent started with two or more drugs within the first month of treatment (ESM Table 3).

**Table 1:** Characteristics of the study population for incidence analysis

Characteristic	All participants	Type 2 diabetes, pharmacologically treated^a^	Type 2 diabetes, non-pharmacologically treated^b^
*n*	3,227,454	36,334	39,162
Sex, *n* (%)			
Male	1,609,058 (50)	22,168 (61)	21,720 (56)
Female	1,618,396 (50)	14,166 (39)	17,442 (45)
Year of birth			
1910–1919	8685 (0.3)	13 (0.04)	15 (0.04)
1920–1929	174,081 (5.4)	1951 (5.4)	3087 (7.9)
1930–1939	261,321 (8.1)	4824 (13.3)	7235 (18.5)
1940–1949	464,082 (14.4)	8955 (24.6)	11,503 (29.4)
1950–1959	592,202 (18.3)	9673 (26.6)	9670 (24.7)
1960–1969	704,276 (21.8)	7489 (20.6)	5492 (14.0)
1970–1979	716,390 (22.2)	3163 (8.7)	2000 (5.1)
1980–1989	306,417 (9.5)	266 (0.7)	160 (0.4)
Education level, *n* (%)			
≤10 years	739,443 (23)	12,904 (36)	13,153 (34)
11–13 years	1,352,663 (42)	16,551 (46)	18,532 (47)
>13 years	1,020,357 (32)	6129 (17)	7017 (18)
No information	114,991 (4)	750 (2)	460 (1)
Place of birth, *n* (%)			
Norway	2,745,774 (85.1)	30,714 (84.5)	34,765 (88.8)
Europe (except Norway)	284,417 (8.8)	2233 (6.1)	1702 (4.3)
Asia	117,438 (3.6)	2303 (6.3)	1921 (4.9)
Africa	44,061 (1.4)	731 (2.0)	513 (1.3)
North and Central America	19,557 (0.6)	158 (0.4)	135 (0.3)
South America	14,012 (0.4)	188 (0.5)	116 (0.3)
Oceania	2195 (0.07)	7 (0.02)	10 (0.03)

Individuals aged 30–89 years in Norway from 2009 to 2014 were included in the analysis

^a^At least one diagnosis of type 2 diabetes and use of non-insulin glucose-lowering medication within 6 months of first being registered with diabetes

^b^At least two diagnoses of type 2 diabetes and not treated with glucose-lowering medication within 6 months of first being registered with diabetes

**Table 2:** Incidence of registered type 2 diabetes in the population aged 30–89 years in Norway from 2009 to 2014 and association of covariates with the risk of type 2 diabetes diagnosis

Characteristic	Incidence cases (*n*)	Person-years	Incidence rate per 100,000 person-years (95% CI)	HR (95% CI)^a^	
				Unadjusted	Adjusted^b^
All participants	75,496	15,463,691	488.2 (484.7, 491.7)		
Male	43,888	7,642,520	574.3 (568.9, 579.7)	1	1
Female	31,608	7,821,171	404.1 (399.7, 408.6)	0.67 (0.66, 0.68)	0.66 (0.65, 0.67)
Year of birth					
1910–1919	28	4327	647.1 (446.8, 937.2)	8.70 (5.62, 13.48)	8.73 (5.63, 13.53)
1920–1929	5038	658,818	764.7 (743.9, 786.1)	6.58 (5.47, 7.91)	6.60 (5.48, 7.95)
1930–1939	12,059	1,282,636	940.2 (923.5, 957.1)	4.14 (3.48, 4.90)	4.15 (3.55, 5.00)
1940–1949	20,458	2,397,076	853.5 (841.8, 865.2)	2.65 (2.26, 3.12)	2.73 (2.32, 3.22)
1950–1959	19,343	3,114,908	621.0 (612.3, 629.8)	1.90 (1.63, 2.22)	1.98 (1.69, 2.32)
1960–1969	12,981	3,708,046	350.1 (344.1, 356.2)	1.53 (1.32, 1.77)	1.57 (1.35, 1.82)
1970–1979	5163	3,644,665	141.7 (137.8, 145.6)	1.27 (1.11, 1.44)	1.29 (1.13, 1.48)
1980–1989	426	653,215	65.2 (59.3, 71.7)	1	1
Education level					
≤10 years	26,057	3,488,171	747.0 (738.0, 756.1)	2.15 (2.10, 2.19)	2.10 (2.05, 2.14)
11–13 years	35,083	6,702,359	523.4 (518.0, 528.9)	1.59 (1.55, 1.62)	1.60 (1.57, 1.63)
>13 years	13,146	4,944,843	265.9 (261.3, 270.4)	1	1
Place of birth					
Norway	65,479	13,565,793	482.7 (479.0, 486.4)	1	1
Europe (except Norway)	3935	1,089,376	361.2 (350.1, 372.7)	1.08 (1.05, 1.12)	1.14 (1.10, 1.17)
Africa	1244	169,999	731.8 (692.2, 773.6)	2.95 (2.79, 3.12)	2.72 (2.57, 2.88)
Asia	4224	486,508	868.2 (842.4, 894.8)	3.15 (3.05, 3.25)	3.08 (2.98, 3.18)
North and Central America	293	86,535	338.6 (302.0, 379.7)	0.88 (0.78, 0.98)	1.02 (0.91, 1.15)
South America	304	57,033	533.0 (476.4, 596.4)	1.75 (1.56, 1.96)	1.83 (1.63, 2.05)
Oceania	7	8447	201.2 (125.1, 323.7)	0.68 (0.42, 1.10)	0.76 (0.47, 1.23)

^a^HRs are from Cox regression analysis

^b^Adjusted for sex, year of birth (in 10-year categories), education level and place of birth

**Fig. 2:**
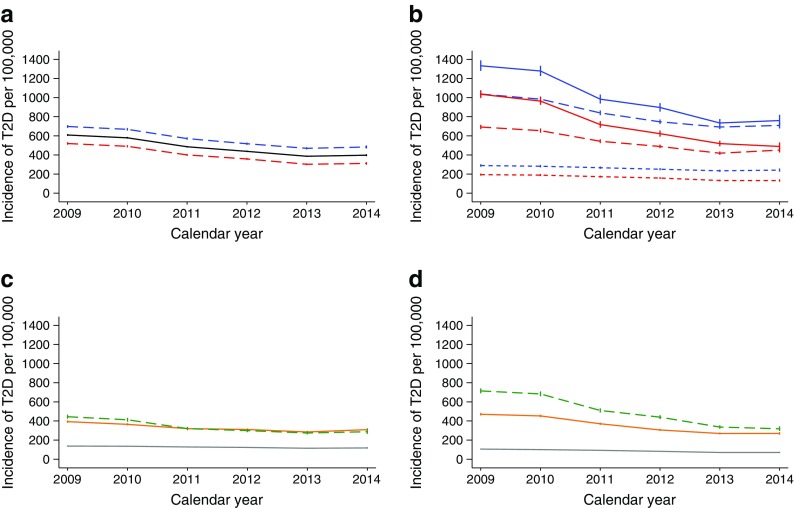
Incidence trends in type 2 diabetes (**a**) Stratified by sex: blue line, men; red line, women; black line, all participants. Average annual per cent change in incidence rate: −10.1 (95% CI −10.5, −9.6). (**b**) Stratified by age group and sex: blue line, men; red line, women; solid line, age group 70–89 years; long dashed line, age group 50–69 years; short dashed line, age group 30–49 years. (**c**) Stratified by age for those treated with glucose-lowering medication within 6 months of diagnosis. (**d**) Stratified by age group for those not treated with glucose-lowering medication within the first 6 months of diagnosis. In (**c**) and (**d**): grey line, age group 30–49 years; orange line, age group 50–69 years; green line, age group 70–89 years. Data are presented as cases per 100,000 person-years with vertical bars representing 95% CI. T2D, type 2 diabetes

During the study period, there was a significant decrease in the incidence of type 2 diabetes in all age groups examined, and we observed a decrease for both pharmacologically treated and non-pharmacologically treated type 2 diabetes (Fig. 2). The incidence of type 2 diabetes decreased from 609 cases per 100,000 person-years in 2009 to 398 cases per 100,000 person-years in 2014. The annual reduction was 10.1% (95% CI −10.5, −9.6). The absolute decrease in incidence was most pronounced in the group aged 70–89, particularly for those not treated with glucose-lowering medication (Fig. 2).

**Fig. 3:**
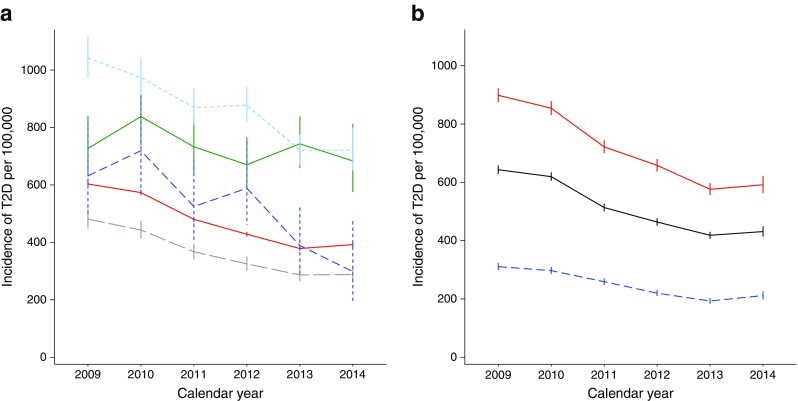
Incidence trends for type 2 diabetes by place of birth and by education level. (**a**) By place of birth: red line, Norway; dashed grey line, Europe (except Norway); green line, Africa; dashed light-blue line, Asia; dashed dark-blue line, South America. Since there were very few cases from Oceania and North and Central America, to improve interpretability, data from these places of birth have not been included in the graph. (**b**) By education: red line, lower education level (≤10 years); black line, medium education level (11–13 years); dashed blue line, high education level (>13 years). Results stratified by education level are shown for individuals born in Norway only (85% of the source population), because of a high proportion of missing data on education level for people born abroad. Data are presented as cases per 100,000 person-years with vertical bars representing 95% CI. T2D, type 2 diabetes

The decline in incidence was statistically significant in all groups except for people born in Africa (Fig. 3a). While the incidence of type 2 diabetes was higher for people with lower levels of education, there was a statistically significant decrease in incidence in all education groups (Fig. 3b). Time trends were similar for men and women (Fig. 2a).

### Change in time trends after recommended use of HbA_1c_ for diabetes diagnosis

We found a small but statistically significant reduction in the rate of change in incidence from September 2012, when HbA_1c_ was introduced as a recommended diagnostic criterion for diabetes in Norway (ESM Fig. 6). While there was a 12.0% decline per year up to this point, the decline in incidence was 7.9% per year thereafter (test for slope change, *p* = 0.001).

### Sensitivity analysis

To investigate whether the observed time trends were sensitive to our operational definition of incident type 2 diabetes, we evaluated whether there were time trends in the number of individuals per year with possible diabetes who did not satisfy our criteria. There were no clear changes in incident unclassified diabetes and in those using non-insulin glucose-lowering medication without having a registered diabetes diagnosis (ESM Fig. 7). These findings indicate that our observed time trends in incidence of type 2 diabetes were robust to different operational definitions.

## Discussion

We observed decreasing incidence of diagnosed type 2 diabetes in Norway during 2009–2014, although prevalence increased at the same time. The declining incidence was seen for both pharmacologically and non-pharmacologically treated type 2 diabetes, and in subgroups defined by age, sex, education level and place of birth.

The main strength of the present study was the use of nationwide registries with nearly complete registration of data and precise linkage by means of the unique personal identification number. We used information on diabetes diagnoses reported from both primary and specialist healthcare, as well as data on dispensing of glucose-lowering medication from pharmacies. The registry-based approach we used relies on clinical judgement and correct coding and reporting [14]. However, we find it likely that we have captured most individuals with diabetes through our pre-defined algorithm for case ascertainment that used combined, longitudinal information from three independent sources.

A major limitation of this registry-based study was that we had no information available on laboratory data. We were therefore not able to formally validate the classification of diabetes based on variables such as islet autoantibodies or C-peptide. Furthermore, some individuals with possible diabetes were inevitably excluded according to our algorithm. Requiring a registered diagnosis of type 2 diabetes, for those treated with non-insulin glucose-lowering medication, could have led to some degree of underestimation of incidence rates. However, the number of individuals per year with incident unclassifiable or potential diabetes (not fulfilling our requirements) was stable over the time period studied, supporting our observation that the declining incidence of type 2 diabetes was not due to an increased number of unclassified cases. The registries used do not register diagnoses or medications received in nursing homes, which may, to some extent, underestimate the incidence and prevalence values in the upper age groups. However, nursing-home residents may also receive primary or specialist healthcare. In addition, the majority of nursing-home residents arrive after a hospital stay (when any diabetes diagnosis should be registered). Only 3.8% of Norwegian residents aged 67–89 live in nursing homes [15] and the average time of residing in nursing homes is only 3.2 years. Thus, missing information on healthcare and medication for people residing in nursing homes is not likely to be a major source of error.

Another possible source of error is misclassification of prevalent cases as incident cases of type 2 diabetes [16]. This could occur for individuals not treated with glucose-lowering medication, or if diabetes diagnoses are not registered because they co-exist with other diseases or conditions. However, if such mechanisms were to contribute strongly to the observed declining incidence, we would expect a much stronger decline from 2009 to 2010 than that from 2010 to 2011, and so forth. As this was not the case, and as we used a relatively long washout period, we consider it most likely that the observed decline in type 2 diabetes incidence was real.

Only a few published studies present recent trends in the incidence of type 2 diabetes. Data from the UK [17] and from Scotland [18], also based on databases, reported stable or decreasing incidence of type 2 diabetes up to 2013. Furthermore, two studies from the USA, one based on claim data [19] and one based on the National Health Interview Survey [20], showed decreasing incidence of diagnosed diabetes in recent years. We are not aware of any previous studies of type-2-diabetes incidence trends that have shown results separately for pharmacologically and non-pharmacologically treated type 2 diabetes. A number of studies have reported trends in the use of glucose-lowering medication, without independent documentation of type 2 diabetes diagnoses. Norwegian data from 2006 to 2011 [3] and Swedish data from 2006 to 2013 [21] both showed a decrease in new users of glucose-lowering medication, despite concomitantly stable or increasing prevalence of diabetes, in accordance with our results.

The Norwegian Health Directorate introduced HbA_1c_ as the primary diagnostic criterion for diabetes in September 2012 [22]. We observed a change in incidence trends thereafter and thus changes in diagnostic practice may have affected trends in incidence.

Our data do not explain why incidence trends have changed. Possibly, some of the observed decline may be a result of an increase in the ratio of undiagnosed to diagnosed individuals in the population after changes in diagnostic activity. General practitioners may also have started using HbA_1c_ in active case finding before 2012, and part of the declining incidence rates may be influenced by this. The introduction of HbA_1c_ as the recommended diagnostic method in 2012 seemed to have a significant, but limited, impact on trends. Furthermore, it is possible that improvements in lifestyle factors may have contributed to some of the observed changes. While the rise in the prevalence of obesity seems to have plateaued in the USA in recent years up, to 2012 [23], it continued to increase in Norwegian adults up to 2008 [24, 25]. Moreover, bariatric surgery for morbid obesity started in Norway around 2004, and approximately 3000 patients underwent such surgery per year during the study period [26]. This may have contributed to the prevention of type 2 diabetes in these individuals, but can only explain a small proportion of the changes in trends. In line with the decreasing trends in the incidence of cardiovascular disease observed in many parts of the world, including Norway [27], the decreasing incidence of type 2 diabetes may be due to changes in lifestyle factors other than obesity. One plausible candidate is the reduction in smoking. In 2006, the proportion of daily smokers among the Norwegian population (aged 16–74 years) was 24%, compared with 12% in 2016. Assuming a constant relative risk of approximately 1.4 for type 2 diabetes among smokers [28], the observed reduction in the prevalence of smokers in Norway during 2006 to 2016 [29] can only account for a small decline in the incidence of type 2 diabetes.

Our analyses of age, sex, education level and place of birth confirmed established type 2 diabetes associations [30, 31]. Importantly, we observed declining incidence rates in all subgroups analysed.

By combining registries, surveillance of type 2 diabetes and planning of appropriate health services can be improved. Incidence and prevalence trends should be considered in combination to assess the burden of diabetes and the challenges to the health system. If the observed trends continue and are confirmed in future studies, it may suggest that some of the public health actions towards improving risk factors for non-communicable diseases are acting effectively. However, further research is necessary to follow future trends, including changes in mortality as a potential explanation for the different time trends in prevalence and incidence of type 2 diabetes.

### Conclusions

Despite the continuing increase in prevalence, we have shown a declining incidence of both pharmacologically and non-pharmacologically treated type 2 diabetes in Norway. This may represent another positive public health trend, in addition to decreasing cardiovascular mortality and increasing life expectancy.

## Electronic supplementary material

ESM (PDF 1196 kb)

## Data Availability

The datasets generated and/or analysed during the current study are not publicly available due to data protection regulations.

## Abbreviations

ICPC-2: The International Classification of Primary Care, Second Edition
NPR: Norwegian Patient Registry
