# Supplementary material for: Decreasing incidence of pharmacologically and non-pharmacologically treated type 2 diabetes in Norway: a nationwide study
Source: Diabetologia. 2018 Jul 11;61(11):2310–8. doi: 10.1007/s00125-018-4681-4 (PMC6182655; doi:10.1007/s00125-018-4681-4)
Supplement: Supplementary file 1 — (PDF 1196 kb) [file 125_2018_4681_MOESM1_ESM.pdf]

Electronic supplementary material (ESM)

Decreasing Incidence of Pharmacologically and Non-Pharmacologically Treated Type 2 Diabetes in Norway 2009-2014

ESM Methods

Interrupted times series model

From September 2012, HbA1c was recommended for type 2 diabetes diagnosis in Norway. We used interrupted time series analysis to evaluate the potential effect of this recommendation on the time trends in incidence of diagnosed type 2 diabetes in Norway. While the model for estimating the overall average percent change per year was a Poisson regression model with only calendar year as a continuous explanatory variable, we evaluated the potential change in level and slope after September 2012 by also introducing 1) a dummy variable for the time period after September 2012 (taking the value zero before Sep 2012 and 1 from Sep 2012 to study end) representing change in level and 2) an interaction (multiplication) term between the dummy variable and the linear term to represent change in slope after September 2012. The model results and predictions are presented in ESM Figure 6.

ESM Tables

ESM Table 1. Time trends, by treatment, in the proportion of people with diagnosed type 2 diabetes aged 30-89 years from 2009 to 2014.

Pharmacologically treated				
**Age groups**	**Year**	**Population**	**Prevalent cases**	**Prevalence (%)**
**Men**				
30-49	2009	770,828	8,570	1.11
50-69		578,191	33,334	5.77
70-89		215,037	20,940	9.74
Total		1,564,056	62,844	4.02
30-49	2010	769,027	9,162	1.19
50-69		588,642	36,684	6.23
70-89		217,562	22,758	10.5
Total		1,575,231	68,604	4.36
30-49	2011	766,599	9,711	1.27
50-69		600,199	39,276	6.54
70-89		219,488	24,066	11.0
Total		1,586,286	73,053	4.61
30-49	2012	763,959	10,188	1.33
50-69		609,623	41,550	6.82
70-89		223,926	25,598	11.4
Total		1,597,508	77,336	4.84
30-49	2013	759,289	10,546	1.39
50-69		617,319	43,402	7.03
70-89		230,340	27,420	11.9
Total		1,606,948	81,368	5.06
30-49	2014	750,424	10,785	1.44
50-69		623,214	44,918	7.21
70-89		239,516	29,773	12.4
Total		1,613,154	85,476	5.30
**Women**				
30-49	2009	710,899	6,370	0.90
50-69		563,503	21,580	3.83
70-89		287,073	22,243	7.75
Total		1,561,475	50,193	3.21
30-49	2010	711,017	6,912	0.97
50-69		573,139	23,516	4.10
70-89		285,690	23,537	8.24
Total		1,569,846	53,965	3.44
30-49	2011	710,212	7,309	1.03
50-69		583,557	25,091	4.30
70-89		284,512	24,314	8.55
Total		1,578,281	56,714	3.59
30-49	2012	709,186	7,652	1.08
50-69		591,864	26,328	4.45
70-89		286,162	25,279	8.83
Total		1,587,212	59,259	3.73
30-49	2013	705,967	7,863	1.11
50-69		599,425	27,425	4.58
70-89		289,270	26,048	9.00
Total		1,594,662	61,336	3.85
30-49	2014	701,206	8,150	1.16
50-69		605,083	28,324	4.68
70-89		295,505	27,107	9.17
Total		1,601,794	63,581	3.97

Non-pharmacologically treated				
Age groups	Year	Population	Prevalent cases	Prevalence (%)
**Men**				
30-49	2009	770,828	2,493	0.32
50-69		578,191	11,069	1.91
70-89		215,037	7,606	3.54
Total		1,564,056	21,168	1.35
30-49	2010	769,027	2,672	0.35
50-69		588,642	12,145	2.06
70-89		217,562	8,408	3.86
Total		1,575,231	23,225	1.47
30-49	2011	766,599	2,768	0.36
50-69		600,199	12,717	2.12
70-89		219,488	8,912	4.06
Total		1,586,286	24,397	1.54
30-49	2012	763,959	2,730	0.36
50-69		609,623	12,885	2.11
70-89		223,926	9,449	4.22
Total		1,597,508	25,064	1.57
30-49	2013	759,289	2,657	0.35
50-69		617,319	12,676	2.05
70-89		230,340	9,708	4.21
Total		1,606,948	25,041	1.56
30-49	2014	750,424	2,465	0.33
50-69		623,214	11,844	1.90
70-89		239,516	9,748	4.07
Total		1,613,154	24,057	1.49
**Women**				
30-49	2009	710,899	2,108	0.30
50-69		563,503	8,430	1.50
70-89		287,073	9,315	3.24
Total		1,561,475	19,853	1.27
30-49	2010	711,017	2,226	0.31
50-69		573,139	9,131	1.59
70-89		285,690	10,074	3.53
Total		1,569,846	21,431	1.37
30-49	2011	710,212	2,327	0.33
50-69		583,557	9,476	1.62
70-89		284,512	10,466	3.68
Total		1,578,281	22,269	1.41
30-49	2012	709,186	2,307	0.33
50-69		591,864	9,539	1.61
70-89		286,162	10,817	3.78
Total		1,587,212	22,663	1.43
30-49	2013	705,967	2,242	0.32
50-69		599,425	9,379	1.56
70-89		289,270	10,946	3.78
Total		1,594,662	22,567	1.42
30-49	2014	701,206	2,079	0.30
50-69		605,083	8,914	1.47
70-89		295,505	10,946	3.70
Total		1,601,794	21,939	1.37

ESM Table 2. Incidence rates per 100.000 person-years of diagnosed type 2 diabetes by sex and age groups for individuals pharmacologically treated and non-pharmacologically treated.

Incidence rates (IR) in pharmacologically treated type 2 diabetes				
**Year**	**Age group**	**No cases**	**Person-years**	**IR per 100,000 person years (95%CI)**
**Men**				
2009	30-49	1175	678915	173.1 (163.5, 183.3)
	50-69	2468	506404	487.4 (468.5, 507.0)
	70-89	919	170512	539.0 (505.2, 575.0)
	Total	4562	1,355,830	336.5 (326.8, 346.4)
2010	30-49	1154	682859	169.0 (159.5, 179.0)
	50-69	2312	513634	450.1 (432.1, 468.9)
	70-89	849	170690	497.4 (465.0, 532.0)
	Total	4315	1,367,183	315.6 (306.3, 325.2)
2011	30-49	1114	687866	162.0 (152.7, 171.7)
	50-69	2098	521626	402.2 (385.4, 419.8)
	70-89	682	170788	399.3 (370.5, 430.4)
	Total	3894	1,380,280	282.1 (273.4, 291.1)
2012	30-49	1097	696615	157.5 (148.4, 167.1)
	50-69	2062	531646	387.9 (371.5, 405.0)
	70-89	638	172738	369.3 (341.8, 399.1)
	Total	3797	1,400,999	271.0 (262.5, 279.8)
2013	30-49	1073	700689	153.1 (144.2, 162.6)
	50-69	2002	537507	372.5 (356.5, 389.1)
	70-89	599	175484	341.3 (315.1, 369.8)
	Total	3674	1,413,680	259.9 (251.6, 268.4)
2014	30-49	545	348601	156.3 (143.7, 170.0)
	50-69	1048	268060	391.0 (368.0, 415.4)
	70-89	333	88799	375.0 (336.8, 417.5)
	Total	1926	705460	273.0 (261.1, 285.5)
**Women**				
2009	30-49	647	648215	99.8 (92.4, 107.8)
	50-69	1541	513280	300.2 (285.6, 315.6)
	70-89	902	239006	377.4 (353.6, 402.8)
	Total	3090	1,400,501	220.6 (213.0, 228.6)
2010	30-49	657	650035	101.1 (93.6, 109.1)
	50-69	1461	520310	280.8 (266.8, 295.6)
	70-89	831	236600	351.2 (328.1, 375.9)
	Total	2949	1,406,945	209.6 (202.2, 217.3)
2011	30-49	606	652472	92.9 (85.8, 100.6)
	50-69	1264	528320	239.2 (226.4, 252.8)
	70-89	614	234127	262.3 (242.3, 283.8)
	Total	2484	1,414,919	175.6 (168.8, 182.6)
2012	30-49	573	657132	87.2 (80.3, 94.6)
	50-69	1263	537696	234.9 (222.3, 248.2)
	70-89	579	234074	247.4 (228.0, 268.3)
	Total	2415	1,428,903	169.0 (162.4, 175.9)
2013	30-49	486	657526	73.9 (67.6, 80.8)
	50-69	1082	543438	199.1 (187.6, 211.3)
	70-89	528	234760	224.9 (206.5, 244.9)
	Total	2096	1,435,724	146.0 (139.9, 152.4)
2014	30-49	252	326202	77.3 (68.3, 87.4)
	50-69	620	270937	228.8 (211.5, 247.6)
	70-89	260	117520	221.2 (195.9, 249.8)
	Total	1132	714659	158.4 (149.4, 167.9)

Incidence rates (IR) in non-pharmacologically treated type 2 diabetes				
Year	Age group	No cases	Person-years	IR per 100,000 person years (95%CI)
**Men**				
2009	30-49	791	678,915	116.5 (108.7, 124.9)
	50-69	2785	506,404	550.0 (529.9, 570.8)
	70-89	1354	170,512	794.1 (752.9, 837.5)
	Total	4930	1,355,830	363.6 (353.6, 373.9)
2010	30-49	771	682,859	112.9 (105.2, 121.2)
	50-69	2741	513,634	533.6 (514.0, 554.0)
	70-89	1333	170,690	780.9 (740.1, 824.0)
	Total	4845	1,367,183	354.4 (344.5, 364.5)
2011	30-49	721	687,866	104.8 (97.4, 112.8)
	50-69	2285	521,626	438.1 (420.5, 456.4)
	70-89	998	170,787	584.4 (549.2, 621.8)
	Total	4004	1,380,280	290.1 (281.2, 299.2)
2012	30-49	650	696,615	93.3 (86.4, 100.8)
	50-69	1909	531,646	359.1 (343.3, 375.5)
	70-89	911	172,738	527.4 (494.2, 562.8)
	Total	3470	1,400,999	247.7 (239.6, 256.1)
2013	30-49	571	700,689	81.5 (75.1, 88.5)
	50-69	1720	537,507	320.0 (305.2, 335.5)
	70-89	690	175,484	393.2 (364.9, 423.7)
	Total	2981	1,413,680	210.9 (203.4, 218.6)
2014	30-49	296	348,601	84.9 (75.8, 95.2)
	50-69	852	268,060	317.8 (297.2, 339.9)
	70-89	342	88,799	385.1 (346.4, 428.2)
	Total	1490	705,460	211.2 (200.8, 222.2)
**Women**				
2009	30-49	614	648,215	94.7 (87.5, 102.5)
	50-69	2013	513,280	392.2 (375.4, 409.7)
	70-89	1572	239,006	657.7 (626.0, 691.1)
	Total	4199	1,400,501	299.8 (290.9, 309.0)
2010	30-49	573	650,035	88.1 (81.2, 95.7)
	50-69	1944	520,310	373.6 (357.4, 390.6)
	70-89	1449	236,600	612.4 (581.7, 644.8)
	Total	3966	1,406,945	281.9 (273.2, 290.8)
2011	30-49	524	652,472	80.3 (73.7, 87.5)
	50-69	1608	528,320	304.4 (289.8, 319.6)
	70-89	1067	234,127	455.7 (429.2, 483.9)
	Total	3199	1,414,919	226.1 (218.4, 234.1)
2012	30-49	468	657,132	71.2 (65.0, 78.0)
	50-69	1366	537,696	254.0 (240.9, 267.9)
	70-89	879	234,074	375.5 (351.5, 401.2)
	Total	2713	1,428,903	189.9 (182.9, 197.1)
2013	30-49	386	657,526	58.7 (53.1, 64.9)
	50-69	1191	543,438	219.2 (207.1, 232.0)
	70-89	691	234,760	294.3 (273.2, 317.1)
	Total	2268	1,435,724	158.0 (151.6, 164.6)
2014	30-49	180	326,202	55.2 (47.7, 63.9)
	50-69	602	270,937	222.2 (205.1, 240.7)
	70-89	315	117,520	268.0 (240.0, 299.3)
	Total	1097	714,659	153.5 (144.7, 162.9)

ESM Table 3. Initial treatment (first month) of newly diagnosed type 2 diabetic patients in Norway 2009-2014. *Incidence estimates until 30th June 2014, to allow ascertainment for 6 months with or without A10 medication, we stopped follow-up time 6 months before end of study period.

Blood-glucose lowering medication	Number of individuals (Total %)	2009	2010	2011	2012	2013	2014*
Metformin	30,024 (82.6)	5,942 (80.6)	6,015 (82.7)	5,403 (84.1)	5,191 (83.6)	4,769 (82.3)	2,704 (82.9)
Sulfonylureas	1,586 (4.4)	602 (8.2)	376 (5.2)	235 (3.7)	175 (2.8)	138 (2.4)	60 (1.8)
Insulin only	340 (0.9)	64 (0.9)	55 (0.8)	46 (0.7)	70 (1.1)	68 (1.2)	37 (1.1)
DPP-4 inhibitors	223 (0.6)	6 (0.1)	24 (0.3)	30 (0.5)	50 (0.8)	63 (1.1)	50 (1.5)
Insulin + Metformin	1,480 (4.1)	262 (3.5)	302 (4.1)	239 (3.7)	283 (4.6)	257 (4.4)	137 (4.2)
Metformin + Sulfonylureas	1,315 (3.6)	389 (5.3)	312 (4.3)	206 (3.2)	193 (3.1)	159 (2.8)	56 (1.7)
Other drugs in monotherapy	96 (0.3)	7 (0.1)	11 (0.2)	21 (0.3)	12 (0.2)	30 (0.5)	15 (0.5)
Other combination therapy (including insulin)	1,270 (3.4)	104 (1.3)	176 (2.4)	245 (3.8)	233 (3.8)	308 (5.3)	204 (6.2)
TOTAL	**36,334**	**7,376**	**7,271**	**6,425**	**6,207**	**5,792**	**3,263**

ESM Figures

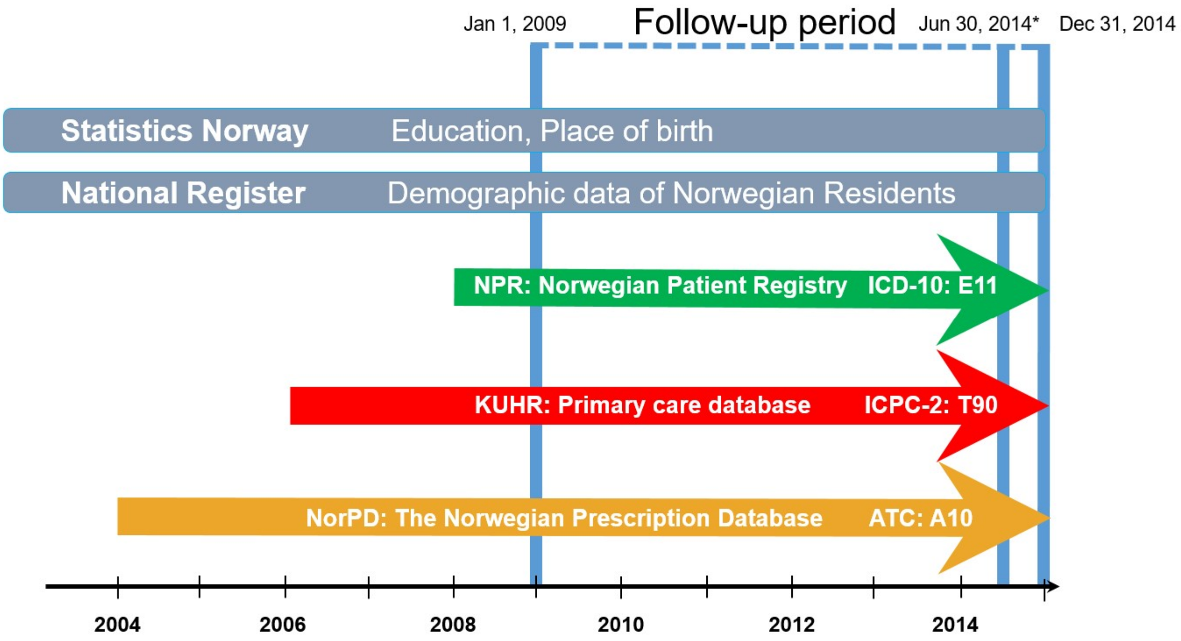

ESM Fig. 1. Registers included in the linkage and their follow up period.

*Data available until December 31st, 2014, to allow ascertainment for 6 months with or without A10 medication in the incidence analysis, we stopped follow-up time 6 months before end of study period.

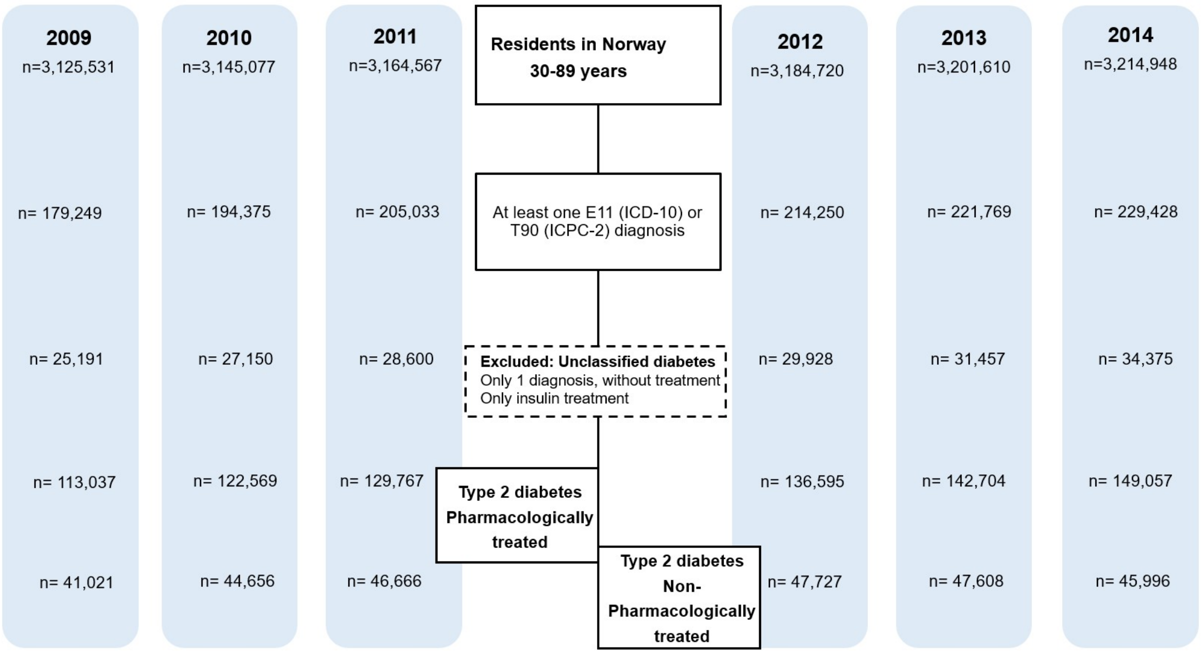

ESM Fig. 2. Flow chart of the study population included in the analysis of type 2 diabetes prevalence. Individuals were diagnosed at any time during the current calendar year or before (and still alive and resident) in the current year.

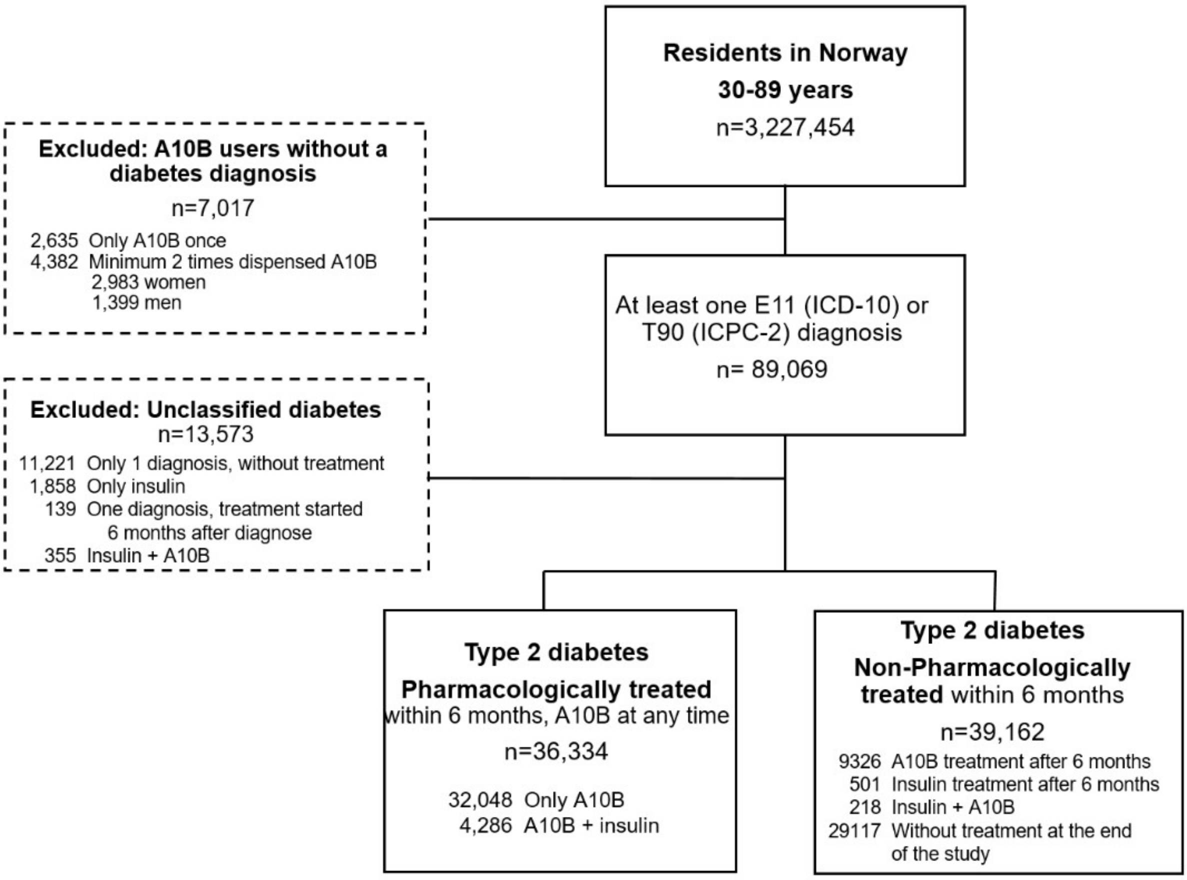

ESM Fig. 3. Flow chart of the study population. This is an open cohort; we follow individuals 30 to 89 years, from January 1st, 2009 until June 30th, 2014. We classified individuals as pharmacologically treated within 6 months after diagnosis (minimum one type 2 diabetes diagnosis and use of glucose lowering medication) and non-pharmacologically treated within 6 months and at least two type 2 diabetes diagnoses. Those not meeting the criteria were excluded as noted “Unclassified”. Some individuals using only non-insulin glucose lowering medication (A10B), but not registered with a type 2 diagnosis, were also excluded.

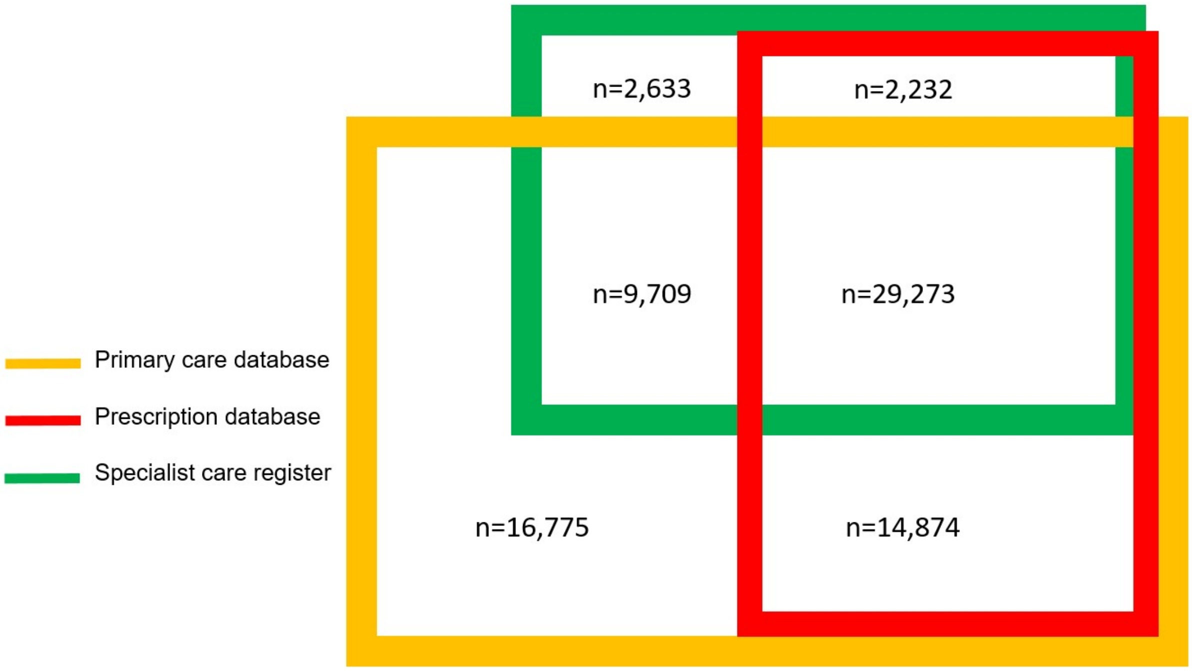

ESM Fig. 4. Number of incident type 2 diabetes cases included in each register, 39% of all incident cases were recorded in the 3 health registers, 36% in 2 registers and 26% in 1 register with at least two registrations of a type 2 diabetes diagnosis.

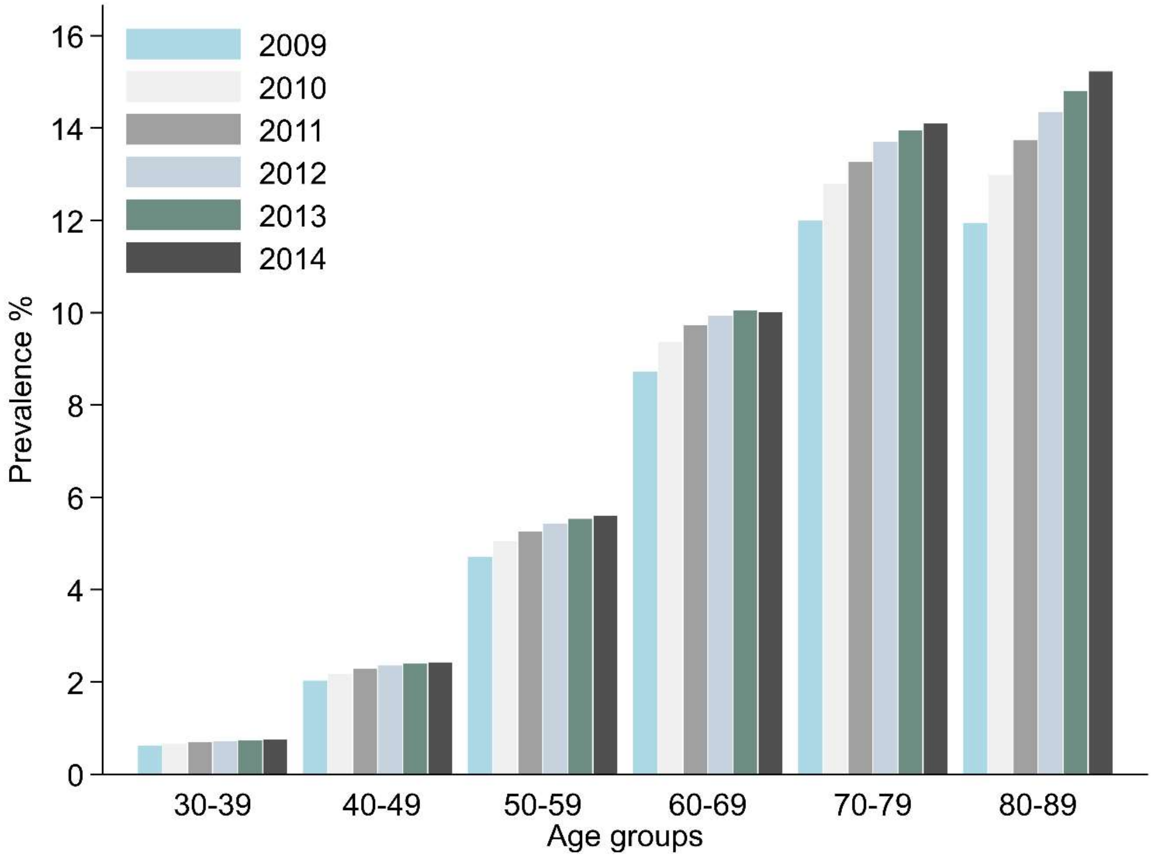

ESM Fig. 5. Prevalence trends in type 2 diabetes from 2009 to 2014, by age groups (years).

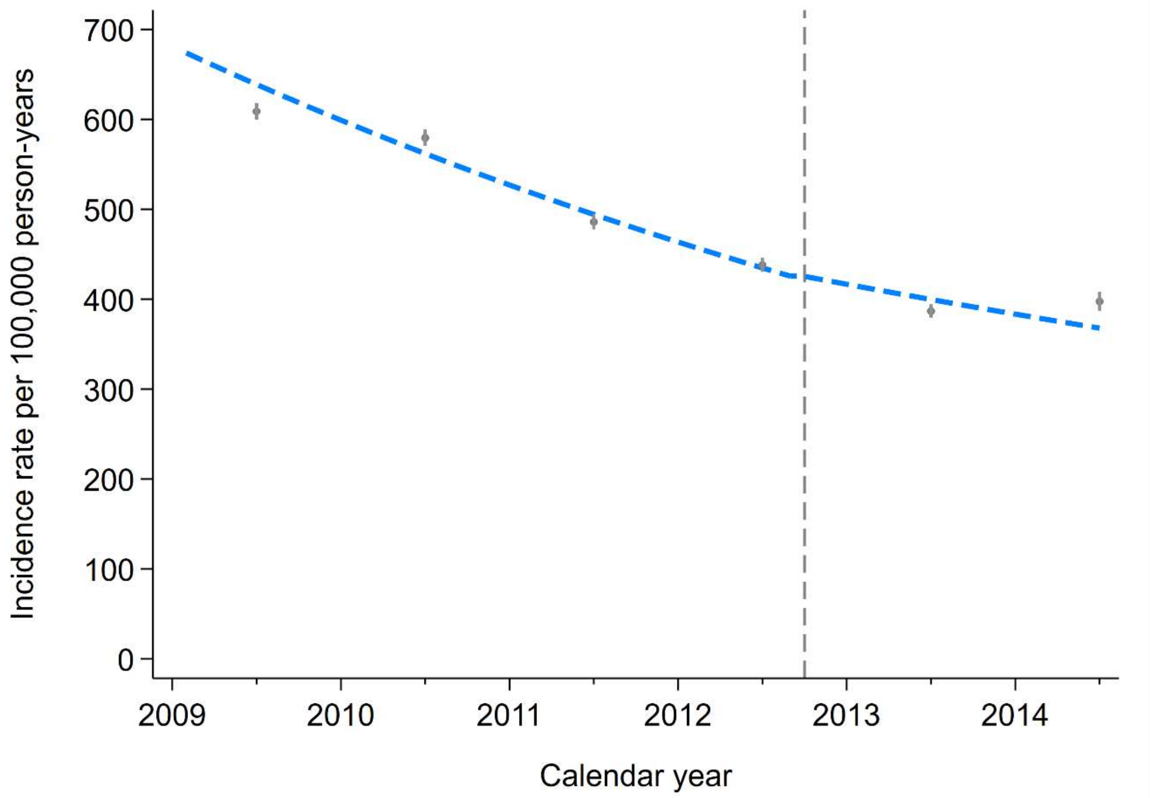

ESM Fig. 6. Time trends incidence of diagnosed type 2 diabetes in Norway before and after HbA_{1c} was recommended for diagnosis of diabetes in clinical practice. From September 2012 (vertical grey dashed line), HbA_{1c} was recommended for diagnosis of diabetes in Norway. There was a significant slope change (p=0.001) from September 2012. From Poisson regression modelling (see ESM methods for details), the estimated decline in incidence rate per calendar year was 12.0% from 2009 to August 2012, and 7.9% per year thereafter. The blue line is the predicted incidence rate from a Poisson model with incidence of type 2 diabetes for each calendar month from 2009 to 2014. The grey dots are observed incidence rates (not based on modelling) in each calendar year with 95% confidence intervals represented by vertical bars (these are placed mid-year on the x-axis).

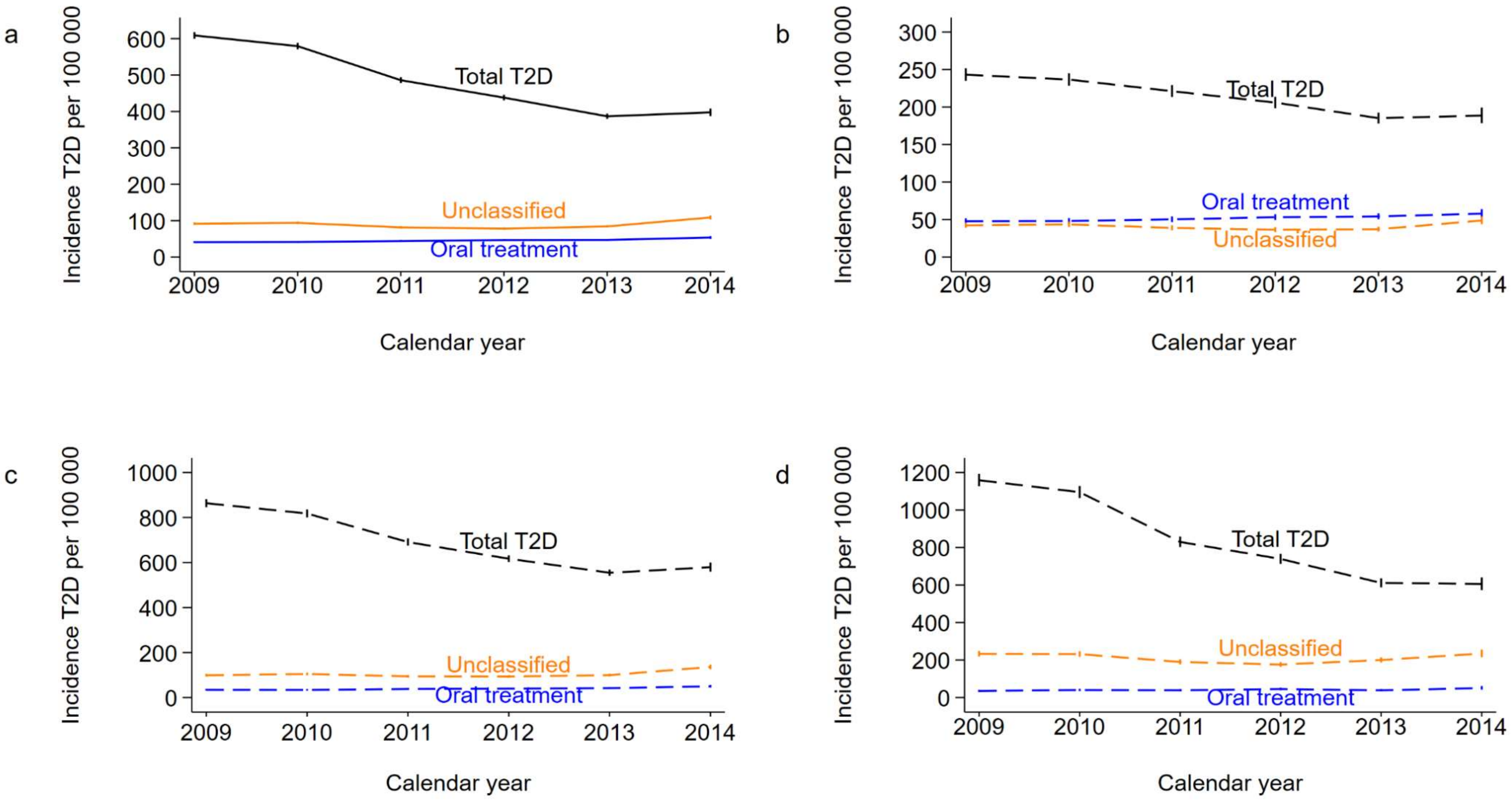

ESM Fig. 7. Sensitivity analysis of misclassification of diabetes. Incidence trends in type 2 diabetes among those included in the study (total T2D in black) and those excluded in our algorithm (unclassified (orange) or those treated with A10B without a registered diabetes diagnosis (blue, denoted oral treatment). a) All age groups, b) Aged 30 to 49 years, c) Aged 50 to 69 years and d) Aged 70 to 89 years. Vertical bars represent 95% confidence intervals.
